# Analysis of Local Track Discontinuities and Defects in Railway Switches Based on Track-Side Accelerations

**DOI:** 10.3390/s24020477

**Published:** 2024-01-12

**Authors:** Susanne Reetz, Taoufik Najeh, Jan Lundberg, Jörn Groos

**Affiliations:** 1 Institute of Transportation Systems, German Aerospace Center (DLR), 38108 Braunschweig, Germany; 2 Department of Civil, Environmental and Natural Resources Engineering, Division of Operation, Maintenance and Acoustics, Luleå University of Technology, 97187 Luleå, Sweden

**Keywords:** railway, switch, acceleration, fault diagnosis, track superstructure, squat, crossing, joint, track-side, way-side

## Abstract

Switches are an essential, safety-critical part of the railway infrastructure. Compared to open tracks, their complex geometry leads to increased dynamic loading on the track superstructure from passing trains, resulting in high maintenance costs. To increase efficiency, condition monitoring methods specific to railway switches are required. A common approach to track superstructure monitoring is to measure the acceleration caused by vehicle track interaction. Local interruptions in the wheel–rail contact, caused for example by local defects or track discontinuities, appear in the data as transient impact events. In this paper, such transient events are investigated in an experimental setup of a railway switch with track-side acceleration sensors, using frequency and waveform analysis. The aim is to understand if and how the origins of these impact events can be distinguished in the data of this experiment, and what the implications for condition monitoring of local track discontinuities and defects with wayside acceleration sensors are in practice. For the same experimental configuration, individual impact events are shown to be reproducible in waveform and frequency content. Nevertheless, with this track-side sensor setup, the different types of track discontinuities and defects (squats, joints, crossing) could not be clearly distinguished using characteristic frequencies or waveforms. Other factors, such as the location of impact event origin relative to the sensor, are shown to have a much stronger influence. The experimental data suggest that filtering the data to narrow frequency bands around certain natural track frequencies could be beneficial for impact event detection in practice, but differentiating between individual impact event origins requires broadband signals. A multi-sensor setup with time-synchronized acceleration sensors distributed over the switch is recommended.

## 1. Introduction

Railway switches are an essential, safety-critical part of the railway infrastructure. Their complex geometry results in increased dynamic loading of the track superstructure by passing trains compared to open tracks. Defects in the track superstructure may require speed reduction zones or, in the worst case, the closure of the line until the track is repaired. On the German rail network in 2022, track superstructure defects on open track and switches were one of the main causes of infrastructure-related disruptions to rail operations [1]. Currently, operators use preventive maintenance strategies with regular, periodically scheduled inspections and repairs, resulting in high costs and sometimes unexpected failures between maintenance intervals. As the deterioration of the track superstructure affects the availability and safety of the switches (and therefore the rail network), continuous monitoring is desirable as it would allow operators to move to condition-based and even predictive maintenance strategies in the future.

A popular method to monitor the track superstructure in general is to measure the accelerations caused by the interaction between vehicles and tracks, either with train-borne or track-side sensor systems [2,3]. In the case of train-borne mounting, large areas of the network can be covered with just a few sensor units, but this requires accurate vehicle positioning. Track-side sensors allow all train passes per switch to be recorded, providing greater volumes of data and a complete picture of the actual load on the switch. Acceleration is often recorded directly at specific critical components (e.g., nose). Network operators can monitor their infrastructure independently, without the need to coordinate with vehicle operators. Both types of acceleration signals simultaneously contain information on track components (rails, sleepers, ballast, etc.) as well as on the train (wheel shapes, bearings, load, etc.). Information on the condition of components whose location allows for good signal transmission (often equivalent to a short distance from the sensor) is more visible in the recorded signals. Any local discontinuity in the contact between wheels and rails, e.g., caused by track defects such as squats or track discontinuities such as joints and crossings, show up as transient impact events in the data [4]. Monitoring for such impact events is important in order to be able to intervene at an optimal time when wear or faults occur (e.g., see [5] for the timing of squat intervention). The characteristics of an impact event depend on its origin and the parameters of the system. One of the main challenges in analyzing such acceleration data is to assign impact events and overall trends as clearly as possible to their origin.

In [4], the authors present a variety of typical dynamic impact loadings caused by wheel–rail interactions and irregularities, with emphasis on the typical shapes of the impact load waveforms. The shape of the impacts varies according to their source, e.g., wheel flats, out-of-round wheels, wheel corrugations, short and long wavelength rail corrugations, dipped welds and joints, pitting and shelling. The high dynamic impact forces along the rails induced by the wheel–rail irregularities can greatly exceed the static wheel load. In all cases, the impact forces are highly dependent on train speed. Loss of wheel–rail contact, known as wheel fly, occurs when the irregularity is large enough or the speed is high enough.

### 1.1. Impact Event Monitoring with Bogie or Axle Box Acceleration Data

In train-borne sensor systems, accelerometers are typically placed on the axle box or bogie [3]. This is an established approach and can be used to monitor various aspects of the track superstructure. The following overview focuses only on transient impact events.

In the Netherlands, a large study was carried out on squat detection using axle box acceleration. The authors of [6] perform a finite element simulation of a squat and a weld and compare the results with measured axle box acceleration data and rail profiles. In [7,8], squats are detected and classified as light, moderate or severe, based on the wavelet power spectrum of the axle box acceleration signal. The method is validated using axle box acceleration signals from measurement trains and data on the severity and location of squats obtained from visual inspection of the tracks. In [9], a three-dimensional finite element model for light and severe squats is developed and validated to capture the dynamic features of axle box acceleration associated with squats in the high frequency range. Based on this model, various influencing factors on the data, including the train speed, the position of the squat in the track relative to the sleeper, and the track design are investigated in [10]. The axle box acceleration measurements are found to be strongly influenced by the train speed. This study also shows that the main frequency characteristics of the axle box acceleration at squats are strongly related to the natural frequencies of the track. This conclusion is confirmed by conducting hammer tests on the track.

In [11], axle box acceleration measurement data are used to derive and verify a squat detection algorithm. Characteristic frequencies and an empirically derived threshold are derived from the wavelet power spectrum of the data. Squats were found to occur mainly in rail sections with rail welds and joints, and sometimes unsupported sleepers. These were shown to influence the characteristic frequencies, but further research is needed in this area.

An exploratory data analysis on axle box acceleration data of insulated joints of different quality is performed in [12]. The insulated joints considered were in good condition, had visible surface degradation of varying degrees, cracks in the fastening or a damaged insulation layer. Several features are proposed for classification, in particular characteristic frequencies associated with the different types of defects. The proposed indicators were verified by means of a hammer test. Based on this information, an algorithm for the detection of isolated squats in good or defective condition with axle box acceleration data is presented and validated in [13].

In [14], the potential of monitoring rail joint bolt tightness using axle box acceleration data is investigated. In a controlled test environment, wavelet scalograms and global wavelet spectra could be used to distinguish between tight, moderately loose and completely loose rail joints.

A vehicle–track dynamic model capable of simulating bogie acceleration on tracks with dipped joints and thermite welds is developed in [15]. In the simulation, feature frequencies obtained from continuous wavelet analysis and feature modes derived from empirical mode decomposition can be used to diagnose track defects. The simulation results are successfully validated with field data.

The ability of axle box acceleration data to monitor crossing degradation is investigated in [16]. The authors compare axle box acceleration data and 3D rail profile measurements on nominal and degraded crossings to extract and verify characteristic vibrations associated with degradation. Axle box acceleration data can be used to identify uneven deformation between the wing rail and crossing nose and local irregularities in the longitudinal slope of the crossing nose. Both defects have characteristic frequency ranges and their severity can be assessed by the energy concentration in these ranges.

The authors of [17] demonstrate a signal processing sequence based on blind signal processing to detect and quantify short track defects in noisy axle-box acceleration data obtained from a shunter locomotive operating in an industrial port railway network. The algorithm separates quasi-continuous band-limited signal components from transient broadband components. The magnitude of the latter reflects the strength of track singularities along the track. Furthermore, local anomalies are detected using traditional signal processing methods and clustered with deep convolutional autoencoders and Gaussian mixture models in axle box acceleration data from the same location in [18]. An optimal number of clusters was found, although the differences between clusters were small. No validation with ground truth data is performed.

The interaction between the vehicle and the track is not only influenced by the state of the track, but also by the state of the train, in particular the wheels. From the perspective of train-mounted acceleration sensors, wheel defects occur periodically as a function of train speed and wheel circumference. This property is used, for example, in [19] to detect wheel defects via cepstral analysis of axle box acceleration data. Similar to track discontinuities or defects, wheel flats create local impact events in the acceleration data. In [20], angular domain synchronous averaging is used to detect wheel flats in axle box, bogie and body acceleration data. The characteristic frequencies of an artificially induced wheel flat in a field test are shown to be speed-dependent, with greater amplitudes at high frequencies at higher train speeds.

### 1.2. Track-Side Acceleration Measurement Systems on Open Track

Unlike switches and crossings, it is not currently possible to comprehensively monitor open tracks (or all critical points such as insulation joints) with track-side sensors due to the number and cost of sensors required. However, there are approaches to using track-side acceleration sensors on straight tracks for train monitoring, especially wheel flats [21,22,23]. When designing track-side sensor systems tailored to switches and crossings, wheel flats and other train-related disturbances will inevitably affect the recorded signals. The results of train monitoring systems can be used to account for these factors in signal processing.

The authors in [24] simulate local discontinuities in wheel–rail contact and their effect on ground vibrations from a pollution perspective, using a numerical vehicle–track–ground railway model. The simulated ground vibration levels are highly sensitive to the height, length and shape of the discontinuities and the train speed. It is found that, for four of the six defect cases, increasing the defect size leads to increased ground vibration levels. The high frequencies generated by the contact forces are rapidly damped by the soil and, to a lesser extent, by the track.

### 1.3. Track-Side Acceleration Measurement Systems Tailored to Switches and Crossings

The integration of accelerometer-based sensor systems into the track superstructure of switches and crossings for condition monitoring purposes is a relatively recent development and the subject of active research.

In [25], a switch is equipped with acceleration sensors, displacement sensors and wheel detectors and train passages are recorded over several months. Based on these data, the author develops a method for monitoring the condition of the substructure and the ballast. The first two natural frequencies of the superstructure are estimated from the measured data using behavioral models. Based on a change in the natural frequencies over time, the degradation of the corresponding components could be monitored. Trains are grouped by type and analyses are carried out separately. The findings suggest that higher train speeds, 150–160 km/h in the case analyzed, are necessary for the superstructure to be sufficiently excited. Considering the same data, the authors in [26] note that, according to a sensitivity analysis, a track-side acceleration sensor can accurately monitor the ballast quality in an area of 10 to 15 m. Thus, a standard 45 to 60 m switch could be covered by three to four sensors.

A selection of signal processing methods specifically tailored to track-side acceleration measurements in switches and crossings is presented in [27]. In particular, both the temporal and frequency characteristics of measured accelerations and their integration into displacements are addressed. The approaches are demonstrated on data from an acceleration sensor mounted on the sleeper that lies beneath the crossing. Using the same sensor setup, in [28] the authors perform an analysis based on a multi-body simulation model with a structural track model and implemented scanned crossing geometries to derive the link between the crossing geometry condition and the resulting track excitation. A crossing condition indicator is proposed, which ignores the quasi-static track response and targets the dynamic track response caused by the wheel–crossing interaction, which is governed by the crossing geometry. After calibration, both the model and the indicator show good results when compared with measured data.

The sensor system presented in [29] is equipped with an acceleration sensor on the switch nose and an inductive unit, which is used to measure the speed of the train. This data can be used to determine the contact position of wheels on the nose. The intended applications for this sensor system are optimization of the geometric profile of the nose and condition monitoring of the nose. For a detailed evaluation of the measurement data, a combination of three physical models is proposed in [30], which represent the relationship between acceleration, dynamic contact forces, wheel–rail contact pressure and wear. In [31,32,33], the authors use advanced signal analysis methods for feature extraction, including Hilbert–Huang transform and wavelet transform, followed by regularization. In combination with wheel contact point and speed, nose health indicators are generated using various regression algorithms. These show a clear correlation with the load (in megatons) on the crossing. The influence of longitudinal acceleration, lateral acceleration and train speed on the health indicator depends on the algorithm, making it difficult to give general recommendations on the selection of features. The same measuring system with an additional camera is used in [34,35] to analyze sleeper settling under load. Large variations in measured accelerations are observed depending on the point of contact and the condition of the switch, even between different axles of the same train and when the switch is in good condition. A correlation between the measured acceleration and weather conditions is verified by simulation, which can partially explain short-term fluctuations in the data.

In the Digiswitch project [36], a railway switch at an experimental test site is step-wise induced with increasingly severe artificial faults (squats and rail wear). Acceleration sensors are mounted on the rods of the switch machine and a bogie is moved over the switch to simulate train passage in a controlled environment. The primary objective of the study was to investigate the feasibility of monitoring wear and other faults of the entire switch system using only accelerometers integrated into the switch machine. According to this project, the integration of accelerometers or other sensor types directly into the turnout machine offers several advantages. The sensor location provides protection from harsh weather conditions and potential damage caused by service vehicles during operations. The data collected from this location can serve a dual purpose, monitoring both the track superstructure and the mechanical components of the point machine. The sensor system can be simplified as the location reduces the need for extensive wiring and packaging, and the power supply can be taken from the point machine itself. However, there is a significant drawback to this approach. The sensors are located at varying distances from the locations of potential track defects and critical track components, such as the crossing. As a result, the signals collected may not always provide sufficient information. In [37], two supervised learning approaches to classify different levels of rail wear in this experimental setup are presented, the first based on spectrograms and residual neural networks, the other based on time domain features and LSTM (long short-term memory) neural networks. In [38], the authors develop a squat detection algorithm for the whole switch based on wavelets, time domain features and isolation forest. Further investigations into squat detection using wavelets are carried out in [39]. However, other transient impact sources such as joints and intersections are also detected without differentiation. The presented algorithms seem to detect impact events (of all kinds), but are not specifically tailored to squats. In this specific experimental setup, squats are the most common local defect in terms of numbers. In general, the experimental setup provides a controlled environment, but it also has some limitations, e.g., very low speed and bogie weight, which influence the results compared to field data.

### 1.4. Contributions

Existing research on impact events in bogie and axle box acceleration data caused by wheel–rail contact discontinuities concentrates on the detection of, e.g., squats or joints on straight tracks, but there is no specific focus on switches. Most publications focus on detection and do not, or only marginally, discuss the diagnosis of different types of impact events. The applications of track-side acceleration sensors in switches have, to date, focused on monitoring the condition of the ballast, uniform rail wear and specific critical components such as the crossing. Apart from an initial investigation into the general detection of impact events in an experimental setup, the monitoring of local track defects and discontinuities has not been addressed, especially the diagnosis of different types of impact event sources.

This paper investigates the different discontinuities in the wheel–rail contact in the experimental setup of the Digiswitch project [36] mentioned above, namely squats, joints and crossings and how these manifest as transient impact events in the measured wayside acceleration. The aim is to understand if and how the origins of these impact events can be distinguished in the data from this experiment, and what the implications for condition monitoring of local track discontinuities and defects with wayside acceleration sensors are in practice. All impact events in the experiment, i.e., the full impact event signature, could be labeled and show repeatable waveforms and frequency content if measurements were taken in the same configuration. The waveform and frequency content of impact events are influenced by a number of factors. The main influence on the impact event signals is the signal transmission from the impact event origin to the sensor, which is determined by their relative locations. Even if all other factors are held constant, it dominates the waveform and frequency content of the recorded signal and overrides the influence of the type of track discontinuity/fault. Due to the small variation in bogie speed and its correlation with other factors, the influence of bogie speed could not be analyzed. No unique frequencies or waveform characteristics could be found for each type of track discontinuity or fault. This is a significant difference to axle box acceleration data, where the transmission between the track discontinuity/fault and the sensor is the same for all impact events, and the frequency content can be used for diagnostic purposes (according to the literature). As previously proposed for axle box acceleration sensors and in the context of this experiment, narrow-band signals around certain natural frequencies of the switch could potentially be used to detect impact events in general. However, signals with a wider frequency bandwidth are required to discriminate between individual events, which in turn could assist in later identification of the type of track discontinuity/fault, i.e., diagnosis. In practice, the impact event signature of a train passing a switch contains a number of overlapping events. It remains to be seen how this affects the detection and diagnosis of impact events. A multi-sensor setup including time-synchronized acceleration sensors distributed over the switch is recommended.

The remainder of this paper is structured as follows. Section 2 introduces the experimental setup, measurement data and methodology for data analysis. Section 3 presents and discusses the results, namely an analysis of the natural frequencies of the experimental setup, the observed impact event signatures, influencing factors on impact events, a detailed view of the squats, joint and crossing and a comparison of all track discontinuities and defects of the experiment together. Section 4 concludes the paper and gives an outlook on future research options.

## 2. Materials and Methods

### 2.1. Experimental Setup

**Figure 1:**
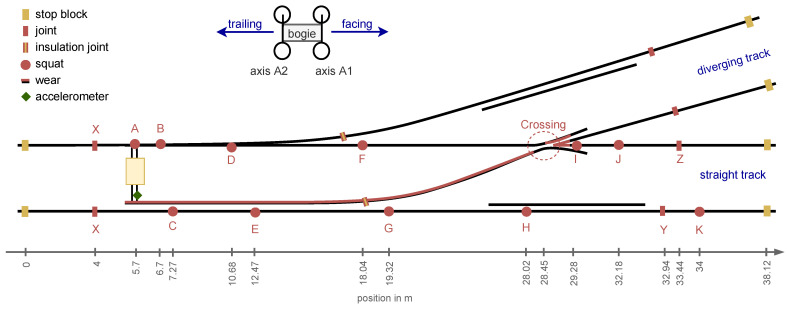
Schematic representation of the experimental setup (not to scale). The squats and joints on the straight track are labeled as A to K and X to Z respectively.

**Figure 2:**
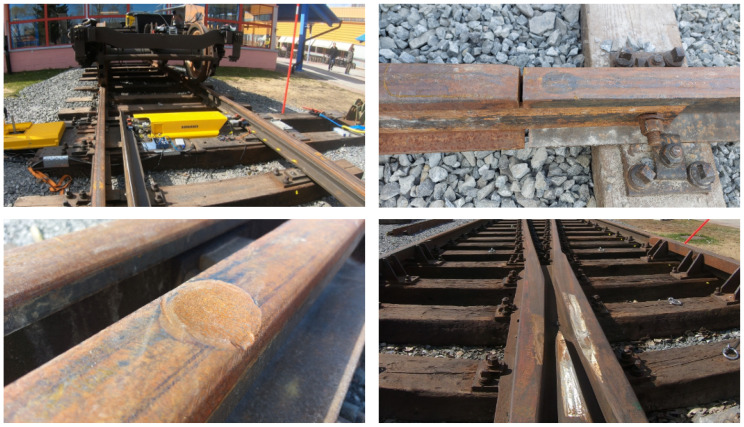
Experimental setup with track discontinuities and defects (cf. Figure 1). Point machine with bogie (**upper left**), joint X (**upper right**), squat D (**lower left**) and crossing (**lower right**).

The experimental setup, shown in Figure 1, consists of a 1:9 railway switch, equipped with track-side acceleration sensors mounted on the rods of the point machine. A bogie is manually pushed over the whole switch from stop block to stop block to simulate train passages on the straight track. On the straight track, there are rail joints, artificially induced squats and the crossing, as seen in Figure 2. The squats have an average depth of 4.1 mm (min 3.7 mm, max 4.7 mm) and an average diameter of 63 mm (min 61 mm, max 66 mm). The crossing is artificially worn out to the point that it is at the end of its lifetime. With the exception of squat A, all squats are located directly above sleepers. By design, the joints are located between sleepers.

Table 1 shows the specifications of the acceleration sensor used in this study. The speed of the bogie is measured by a tachometer on the first axle. More details on the data acquisition can be found in [37].

### 2.2. Measurement Data

The raw measurement data (started before the bogie starts to move and stopped after it hits the stop block at the other end of the switch) is pre-processed, using the following steps:

1. Detrend and demean signal.
2. Apply Tukey window of length 2 s at the beginning and the end of the time series.
3. Apply Butterworth bandpass filter (forward and backward) with filter frequencies 1 to 8000 Hz and filter order 2.
4. Cut the signal down to where the bogie is moving from stop block to stop block. This also removes the areas affected by the Tukey window at the head and tail of the measurement.
5. Down-sample by 2 to a sampling frequency of 25,600 Hz.

The literature on impact loading in railways [4] and on axle box acceleration places frequencies of interest mostly below 2000 Hz. The used sensor does provide a flat frequency response between 1 and 8000 Hz, with low-frequency oscillations and high-frequency noise outside of this range. The pre-processing thus filters the data accordingly and reduces the relatively high sampling rate.

In later sections, the measurement is filtered to frequency bands of narrower bandwidth for specific applications involving only short sections of data around impact events. Here, the window and filter steps are reapplied to the entire measurement time series from stop block to stop block, with the filter frequencies set to the respective narrower frequency bands mentioned.The data around the impact events are then extracted. With this configuration, the Tukey window does not affect the data of interest as the impact events are some distance from the stop blocks.

**Figure 3:**
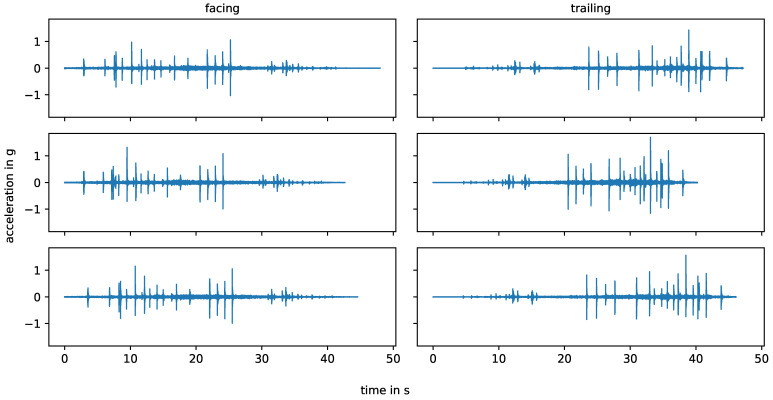
Pre-processed measurements obtained from stop block to stop block on the straight track.

Figure 3 shows the six bogie passes from stop block to stop block on the straight track, measured by one vertical (skywards) acceleration sensor, that are considered in this study. Three measurements are in the facing direction, while the other three are in the trailing direction. The different lengths of the measurements are due to variations in the bogie speed, which remains below 1.2 m/s for all measurements.

### 2.3. Methods

#### 2.3.1. Frequency Analysis

In frequency analysis [40], the energy spectral density describes the frequency content of signals whose energy is concentrated around a finite time interval or whose total energy is finite, such as pulse-like signals. Thus, the transient impact events in the experimental data are analysed using periodograms, i.e., the modulus square of the Fourier transform, to estimate their energy spectral density. In contrast, the power spectral density applies to signals that exist over all time or over periods of time long enough to be considered infinite. It refers to the spectral energy distribution that would be found per unit time if the total energy were infinite. To reduce the variance in the spectrum at any given frequency, time-averaging approaches such as Welch’s method can be used instead of periodograms if a larger number of samples are available and the signal can be assumed to be a stationary stochastic process. This method is used in this paper to analyze the frequency content of the whole measurements from stop block to stop block.

#### 2.3.2. Waveform Analysis

There are several options to measure the similarity of the shape of time series in the time domain, e.g., Euclidean distance, mean absolute percentage error, dynamic time warping, or (normalized) cross-correlation. If an exact match of the starting times of the time series cannot be guaranteed, it is desirable to allow for a fixed time lag between the two time series without penalizing similarity.

The Pearson correlation coefficient [41,42] of two uniformly sampled time series *x* and *y* is defined as the sample covariance over the product of their sample standard deviations. Applying zero-padding $y={\left(\ldots ,0,y_{1},\ldots ,y_{n},0,\ldots \right)}^{T}$, the correlation coefficient between *x* and *y* for time lag *l* is defined as

$$\begin{array}{c} \rho_{x,y}^{l}=\frac{\sum_{t=1}^{n}\left(x_{t}-\bar{x}\right)\left(y_{t+l}-\bar{y_{l}}\right)}{\sqrt{\sum_{t=1}^{n}{\left(x_{t}-\bar{x}\right)}^{2}}\sqrt{\sum_{t=1}^{n}{\left(y_{t}-\bar{y}\right)}^{2}}},\;\bar{y_{l}}=\frac{1}{n}\sum_{t=1}^{n}y_{t+l}\;. \end{array}$$

The correlation coefficient has values between −1 (total negative linear correlation) and 1 (total positive linear correlation), where 0 indicates no correlation. Due to the normalization, the correlation coefficient is independent of signal amplitudes. Given a time lag bound, maximizing the correlation coefficient determines the optimal time lag between two series with respect to linear correlation. To compensate for the zero-padding and thus non-symmetry, both the coefficients for x,y as well as y,x are considered.

When applied to acceleration time series, the correlation coefficient is mostly affected by high amplitude frequencies and results may suffer from broadband signals. Therefore, acceleration data are typically band-pass filtered to the frequency range of interest beforehand.

## 3. Results and Discussions

### 3.1. Frequency Analysis

**Figure 4:**
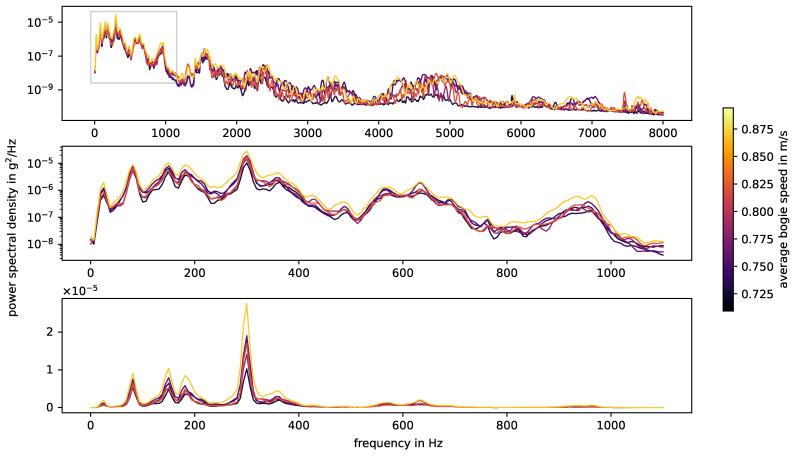
Power spectral density of whole acceleration measurement series, colored by average speed (calculated over the whole speed measurement series). The number of samples per window in the calculation of Welch’s method is 4096 and the windows overlap by 2048 samples. The lower two plots zoom in on the frequency range up to 1100 Hz, with densities displayed in log and linear scale, respectively.

Figure 4 shows the power spectral densities, calculated using Welch’s method, for all measurement series from stop block to stop block. Below about 1100 Hz, the spectra of the different measurements are very similar. Peaks are located at approximately the same frequencies, namely at 25 Hz, 80 Hz, 150 Hz, 180 Hz, 300 Hz, 565 Hz, 630 Hz and 940 Hz. The measurement with consistently higher amplitudes in this frequency range also has the highest average speed. The overall speed variations are small and do not systematically affect the excited frequencies.

There are three main natural frequencies in open rail tracks (cf. e.g., [4,43,44,45]), whose values depend on track parameters:

- In-phase or rail and sleeper (as a mass) on ballast vibration: 40–200 Hz;
- Out-of-phase or rail on railpad vibration: 200–670 Hz;
- Pin-to-pin, i.e., bending wave of the rail with wavelength twice the sleeper spacing: 600–1500 Hz.

Determining the exact track frequencies can be challenging. The author of [43] points out that, for some calculation models, the measured pin-to-pin frequency lies in the range of 10–300 Hz beneath the calculated one. The interpretation of measured frequencies is not entirely unanimous in the literature. The authors in [10] add full track resonance to the list of main natural frequencies. They observe full track resonance at 100 Hz, sleeper anti-resonance at 280 Hz, rail resonance at 1008 Hz and pin–pin resonance at 1150 Hz. For switches and axle box acceleration data, the frequencies observed by hammer tests in [46] do not exactly match up with the natural frequencies of the track observed in ABA data. The authors contribute this to the loading conditions of the track, for the axle box acceleration the track is pre-loaded which is not the case for the hammer tests. They also point out that “this simplified setup is appropriate for plain tracks, but is less useful at crossings due to their complex structure and discontinuous geometry. This implies that the measured vibrations may differ significantly at each sensor position, leaving the dynamic behavior of the entire structure unclear”.

In the experimental setup of this paper, the sensor is attached at the rod of the point machine by a steel casing and glue. The point machine rod is connected to the switch blades by soft pads and screws. This setup itself has natural frequencies that are unknown and are likely to affect the measurement data. The in-phase vibration would be among the lower frequencies 25 Hz, 80 Hz, 150 Hz and 180 Hz. The out-of-phase vibration would be one of 300 Hz, 360 Hz, 565 Hz and 630 Hz, of which 300 Hz is the most prominent frequency. The pin-to-pin frequency strongly depends on the sleeper distances [43]; the smaller the distance between the sleepers, the higher the pin-to-pin frequency. In the experiment, the sleeper distances vary with a median value of 0.7 m, which is average compared to the literature. In general, uneven sleeper spacing dampens the power of the pin-to-pin frequency. The pin-to-pin frequency for wooden sleepers is higher than for concrete sleepers due to their lower mass. Following the literature, the peak at 940 Hz in the experimental data is most likely related to the pin-to-pin vibration.

**Figure 5:**
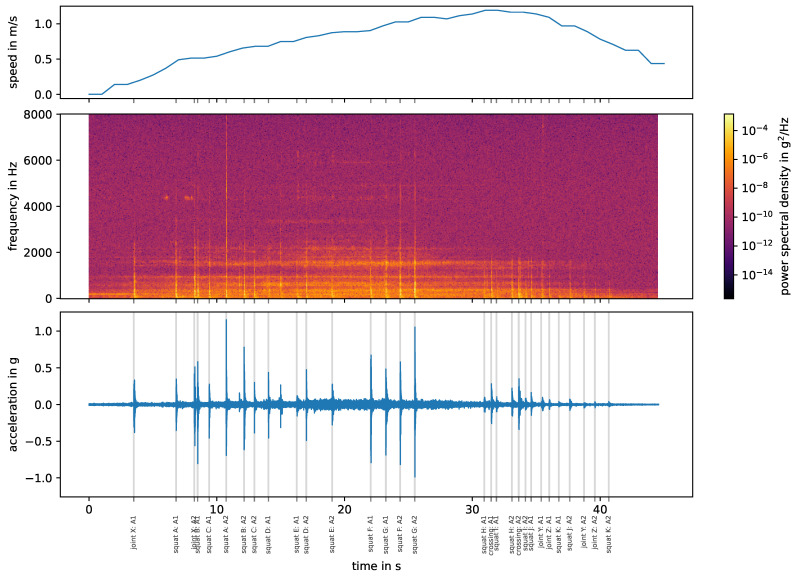
A single measurement on the straight track in facing direction. All known impact events according to Figure 1 are labeled (“impact event origin: axle”) at their start times.

Vertical and lateral waves that propagate along a rail track decrease in amplitude with distance from their excitation point. This effect is quantified by the track decay rate, which can be determined by means of theoretical calculations, hammer tests or train passages [47]. The presence of a train and thus a pre-loading of the track influences the measured track decay rate [48]. Generally, the better the track damping, the faster the rail vibration decays. On open straight tracks, the track decay rate can be used to improve the interpretation of signals originating at different distances from a sensor. In the case of switches, this is more complicated. In the experimental setup, the sensor is mounted on the rods of the point machine and the bogie runs over the straight track (cf. Figure 1). The best transmission from impact event to sensor seems to be in the area of the intermediate rails (middle of the switch), where the bogie is directly riding on the switch blade that is connected to the point machine rods and thus the sensor, as seen in Figure 5 (middle plot). Here, the bogie is at its highest speed when it reaches the crossing, but the following impact events have much lower amplitudes than those in the intermediate rail area. Generally, due to the complex geometry of the switches, the effect of the location of a signal’s origin relative to the sensor on the signal transmission cannot easily be broken down to the distance and a universal track decay rate. To experimentally determine the signal decay rates in a switch for a given sensor position, hammer tests would have to be performed over the entire switch, on each rail, at very close intervals. The measured signal decay could then be mapped over the layout of that particular switch. Such tests are not available for the experimental setup described in this paper. Performing such tests over a range of switches and for different sensor positions could help to understand more about track decay in switches in general. Another option would be to compare the measured data with a simulation of the entire turnout to better understand both the natural track frequencies and the track decay rates. However, the complex layout of the experimental setup would make such an approach very challenging.

### 3.2. Impact Event Signature

In the following, a complete labeling of when which axle hits which track discontinuity/fault is called an impact event signature (cf. Figure 5, bottom plot). Given the layout of the switch with the location of joints, squats and the crossing, as well as the bogie speed and thus the position, an impact event signature for each measurement can be calculated. To account for measurement inaccuracies, the impact events are manually re-labeled, assisted by blind signal separation as a peak detection method (see [17]). The impact event signatures are reproducible across all six measurements. All pre-calculated impact events could be labeled and there is no overlap between events. While joints and squats generally appear as a single impact in the data, the crossing consists of two successive impacts per wheel. Due to the fact that joint X is present on both rails (double joint), there are also two impact events with a very short time offset (sometimes not even visible). The impact events caused by squats and joints have a approximate duration of 0.1 s, the crossing of 0.2 s. These values are used for all further calculations.

**Figure 6:**
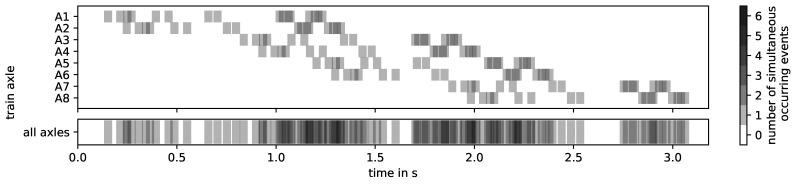
Hypothetical impact event signature of an 8-axle passenger train (Bombardier Regina X52) over the experimental switch with all its track discontinuities and defects at 100 km/h. Squats and joints are assumed to have a signal duration of 0.04 s (value adapted from [8]), and the crossing is 0.08 s.

The impact events in the presented experiment are clearly separated from each other in time. This is not necessarily the case in practice (cf. Figure 6), as the number of impact events increases with the number of axles and the time between events is shorter. In [8], impact events in axle box acceleration caused by squats have a duration of 0.04 s at a train speed of 100 km/h. Therefore, it is safe to assume that the duration of track-side measured impact events does not decrease sufficiently with higher train speeds to prevent the overlapping of events, if at all.

### 3.3. Influencing Factors on Impact Events

A local discontinuity in the wheel–rail contact results in an impact event, some distance from the sensor. At the sensor, we measure the signal from the impact itself, as it is transmitted through the system to the sensor, and the natural frequencies of the system which are excited by the impact. The studied impact events vary in their type (joint, squat, crossing), which directly influences the source signal of impact events. The artificial squats in the experimental setup are more consistent in their shape as real squats in practice, and the joints Y and Z are comparable in their shape as well. Other than the impact event origin type, the following factors are to be considered:

- Location of impact event origin with respect to sensor: The location dominates the transmission of the signal toward the sensor and the natural frequencies of the system that are excited by impact events. A main (but not isolated) factor is the absolute distance between the impact event origin and the sensor.
- Driving direction: Facing or trailing. This factor directly changes the geometry of the impacts.
- Axle: A1 or A2. While no vehicle faults were noted, the axle (especially the wheels) themselves differ from each other. This directly changes the source excitation of the impacts. Furthermore, depending on its location, the axle not involved in an impact event influences the transmission between impact origin and sensor and the natural frequencies of the system.
- Bogie speed: Influences the strength of impact events. Theoretically, higher speeds can also change the geometry of impact events (cf. [4]) due to a loss of contact between wheel and rail, but this is not the case in the experiment.

The experimental setup offers little space for accelerating and braking the bogie, such that, for the track discontinuities and defects at the beginning and end of the switch, the bogie speed is correlated with the measurement direction and the axle. The data do not contain enough speed variations to properly investigate the influence of bogie speed on impacts while all other influencing factors remain constant.

**Figure 7:**
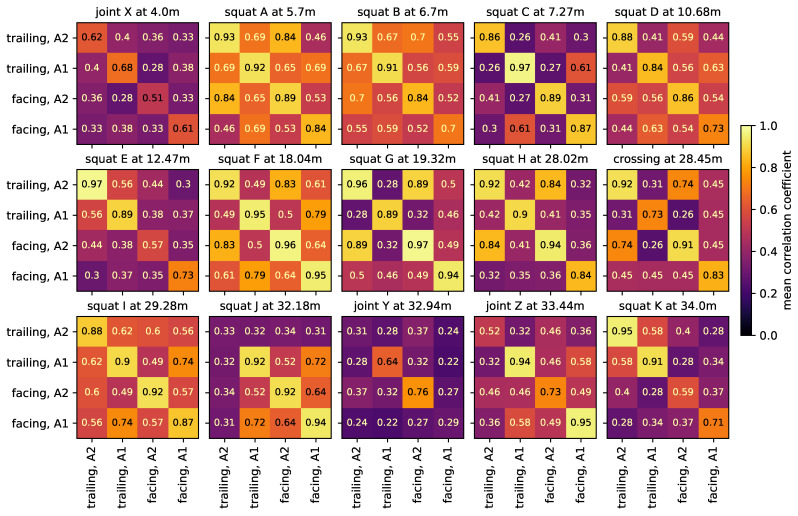
Mean correlation coefficient between impact event time series of different directions and axle for each impact event origin. The data are band-pass filtered to 10–1000 Hz and the maximal allowed timelag is set to 0.01 s, to compensate for the manually labeled start times.

Figure 7 shows the mean correlation coefficients between impact event time series of different directions and axles for each impact event origin in the experiment. In each plot, the diagonal contains the average of correlation coefficients between different impact events with the same driving direction and axle. For each impact event origin, there is also little variation in bogie speed between impacts of a given direction and axle. Overall, these values are consistently high (mean 0.82), indicating that the waveform of impact events caused by the same origin remain similar if all other factors remain the same. The non-diagonal elements are the average of correlation coefficients between impact events with two different combinations of driving direction and axle. Here, the bogie speed can vary slightly more. For some combinations, the correlation coefficients are still relatively high, but often, this is not the case (mean 0.47). This shows that, if at least one factor changes, the waveforms can vary drastically. All these observations also hold true for the energy spectral densities.

**Figure 8:**
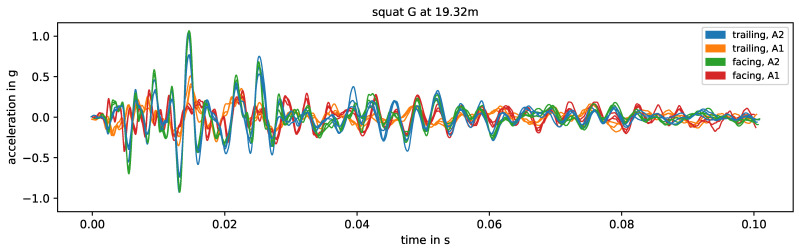
Measurement data of squat G, colored by direction and axle. The data are band-pass filtered to 10–1000 Hz after standard pre-processing and aligned using the timelag derived from the correlation coefficient (maximal allowed timelag set to 0.01 s).

Figure 8 shows the measurement data of squat G in the intermediate rail area, colored by direction and axle. For the same direction and axle, the waveform is very similar. The same holds true for both directions of axle A2, but not as strongly for both directions of axle A1. These observations are reflected in the mean correlation coefficients in Figure 7. For this squat, the speed changes very little for all combinations of direction and axle and has no relevant effect on the waveform.

**Figure 9:**
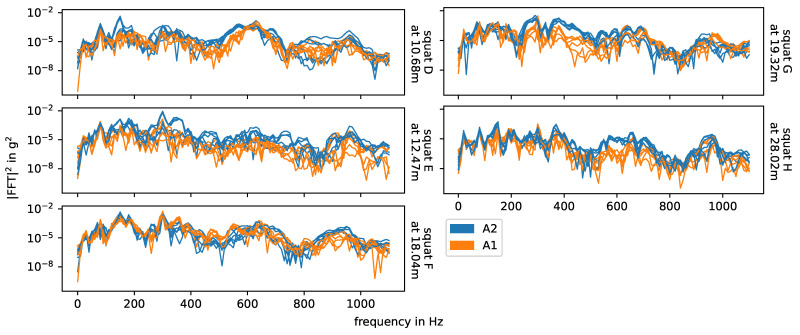
Energy spectral densities of squats in the area of the intermediate rails on a logarithmic scale, colored by axle. Axle A2 is in between axle A1 and the sensor.

For the squats in the area of the intermediate rails, especially the axle seems to have a strong impact on the correlation between the impact event data. Figure 9 shows the respective energy spectral densities of all measurements, colored by axle. For certain frequency ranges between 200 Hz and 1000 Hz, there are systematic differences between the energy spectral densities related to the two axles, but the effect changes for every squat. Generally, another axle between the sensor and the impact event origin changes the transmission of the signal. The rails between the sensor and the impact event origin are pre-loaded. Here, this is the case for axle A1, but not for axle A2 (independently of the driving direction). Recalling the previous discussion of the natural frequencies of the switch in the experiment, the higher frequencies between 200 Hz and 1000 Hz include rail on railpad and pin-to-pin vibrations, while the lower frequencies below 200 Hz include the rail and sleeper on ballast vibration. A load on the track should have a greater effect on the first two than on the latter. However, different frequencies have different transmission rates through different media, so the change in system configuration from one axle to another may affect the transmission rate for each frequency differently.

### 3.4. Squats

The impact events caused by a single track discontinuity/fault show a significant number of variations in their waveforms and frequency content depending on various influencing factors. For a detailed comparison between different impact event origins, we keep these factors as constant as possible.

**Figure 10:**
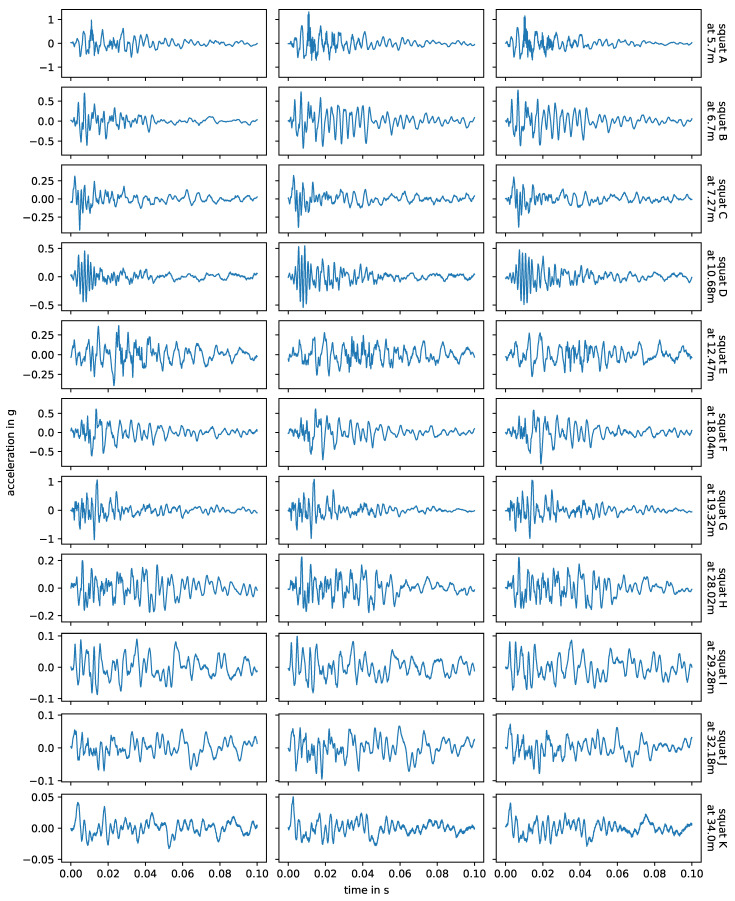
Squats observed in facing measurements, with axle A2. Each column corresponds to one measurement (i.e., one bogie passage over the switch), each row to a specific squat. The data are filtered to 1–8000 Hz during pre-processing.

**Figure 11:**
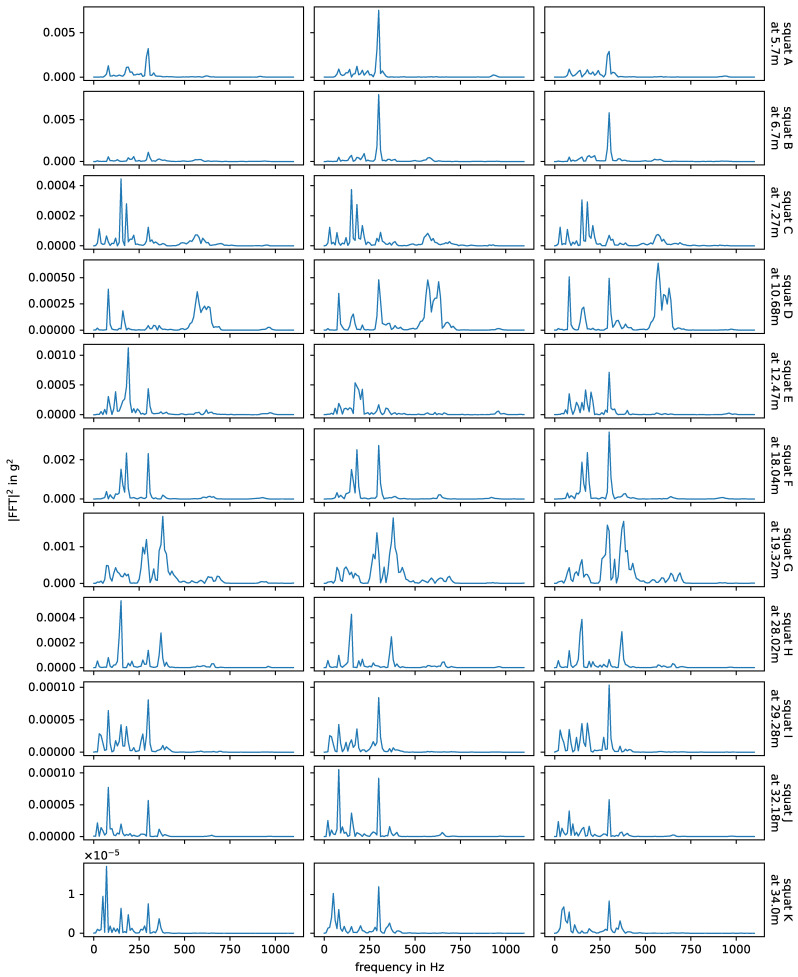
Energy spectral densities of squats observed in facing measurements, with axle A2. Each column corresponds to one measurement (i.e., one bogie passage over the switch), each row to a specific squat, as in Figure 10.

Figure 10 shows the impact events caused by squats and axle A2 in measurements with facing driving direction, and in Figure 11 we see the corresponding energy spectral densities. In this case, the bogie is moving away from the sensor and the impact events are caused by the axle closer to the sensor, i.e., there is no other axle in between the impact origin and the sensor. The bogie speed varies little between the three measurements of each squat, but it is not constant over the whole switch and may affect the plot. The relation between amplitude and distance to sensor is non-linear, as already discussed at the end of Section 3.1. The squats H, I, J and K are farthest away from the senors and do have significant lower amplitudes. For each individual squat, the waveforms of all three measurements (i.e., one row in the plot) are nearly the same and the energy spectral densities are also very similar, but there are huge variations between squats. The waveforms in particular are very distinct and unique. Squats that are located closely to each other are more similar than those further apart, e.g., squats A and B and squats J and K. The frequencies observed for the squats line up with the natural frequencies of the experimental setup discussed in Section 3.1 and shown in Figure 4. The overall most prevalent one is approximately 300 Hz, though this is not the case for all squats.

In the literature on axle box acceleration data, the squats in [7,8,9] show two frequency peaks, one around 300 Hz and one in the range of 1000–2000 Hz, with some variations. Lighter squats excite higher frequencies than moderate-to-severe squats. Here, the squats are very consistent in their frequency content. Due to their artificiality (see Figure 2), the squats in the experiment are already more consistent in their shape than squats in practice. Still, the experiment shows that, with a wayside sensor system, a minor change in location relative to the sensor results in significant waveform and frequency content changes for squats in switches. This can have a strong impact on squat detection and diagnosis methods, as there is no specific characteristic that can be attributed uniformly to all squats.

### 3.5. Joints

**Figure 12:**
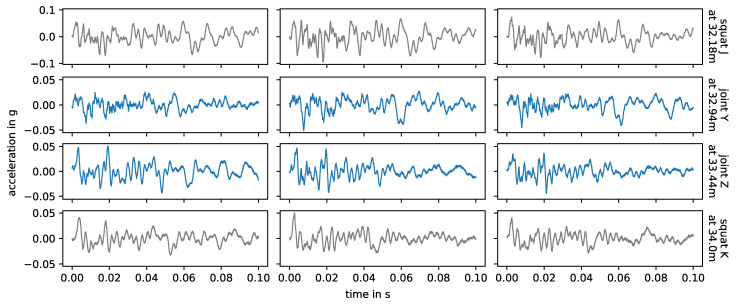
Joint Y and Z (blue) and adjacent squats (gray), observed in facing measurements, caused by axle A2. Each column corresponds to one measurement (i.e., one bogie passage over the switch), each row to a specific joint or squat, as in Figure 10. The data are filtered to 1–8000 Hz during pre-processing.

**Figure 13:**
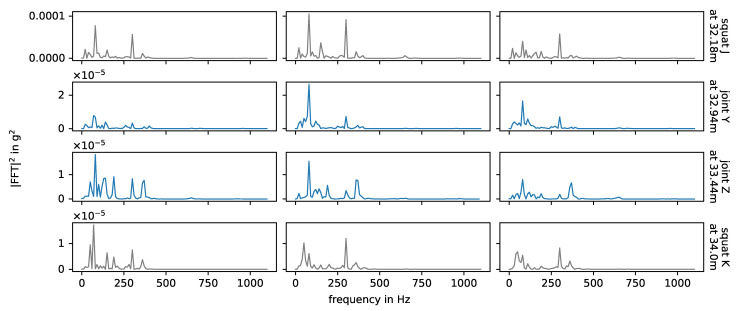
Energy spectral densities of joint Y and Z (blue) and adjacent squats (gray), observed in facing measurements, caused by axle A2. Each column corresponds to one measurement (i.e., one bogie passage over the switch), each row to a specific joint or squat, compared to Figure 11.

Figure 12 shows the impact events caused by joint Y, joint Z, their adjacent squats and axle A2 in measurements with facing driving direction. The corresponding energy spectral densities are displayed in Figure 13. Again, the waveforms and observed frequencies are very similar for the same joint, but there are notable differences between both joints. Comparing these with Figure 10 and Figure 11, squats and joints in the same area have more similar waveforms and frequencies than squats in different areas of the switch. In [6], the authors simulate a squat and a weld with a finite element model and compare the results with exemplary field test axle box acceleration data up to 1000 Hz. The different geometries of the squat and the weld in the simulation do result in different simulated frequency responses in the axle box acceleration and both are in good agreement with the field test data. The squat is placed close to a sleeper while the weld is positioned between sleepers, which may influence the results. Generally, separating out different types of track discontinuities or defects by their frequency content seems to be a possible approach for axle box acceleration data. This is not the case in the data of this experiment, as the location relative to the sensor is a much stronger influence factor than the type of track discontinuity or fault.

Joint X is a double joint, i.e., both rails have a discontinuity at the same position. In the experimental data, the wheels of one axle of the bogie do not always hit the rail discontinuities at the exact same time, causing an overlap of two impact events with a varying short time lag in between. This is potentially also the reason why, as seen in Figure 7, impact events caused by joint X have low correlation coefficients, even for the same set of influencing factors.

### 3.6. Crossing

**Figure 14:**
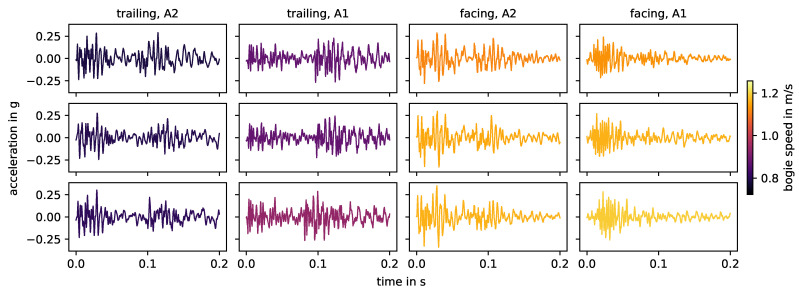
Impact events caused by the crossing. The data are filtered to 1–8000 Hz during pre-processing.

**Figure 15:**
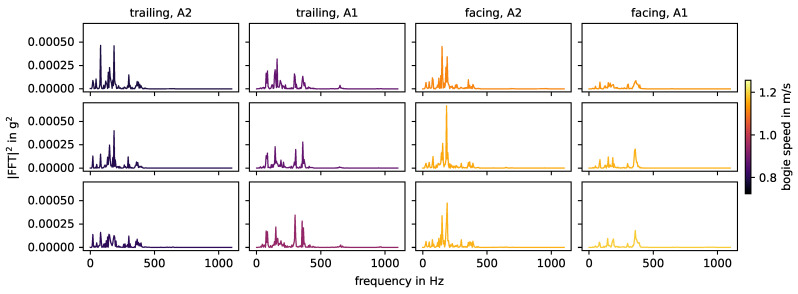
Energy spectral densities of impact events caused by the crossing, compared to Figure 14.

In the experiment, the crossing causes impact events with a characteristic double peak, as seen in Figure 14. In the facing direction, the double peak is only clearly visible for the axle A2, for reasons that are unclear. For axle A1, the waveform has the shape of a single peak, but much broader than the peaks related to squats in the same area. When measured at a distance, these waveforms are prone to misinterpretation as two individual peaks from different sources that occur shortly after each other or are overlapping. The energy spectral densities of the impacts in Figure 15 again show the natural frequencies of the switch. For both the acceleration and the energy spectra, the variations in bogie speed do not correlate with changes in the magnitude of the amplitudes. The speed varies less than 0.4 m/s, so this factor cannot be properly investigated here.

For condition monitoring of local track discontinuities that are spread out over a switch (e.g., joints, welds) and defects (e.g., squats, studs), the crossing plays an important role in the sense that it is part of the impact event signature. The crossing itself is an expensive safety critical component that underlays very high dynamic loading due to its design. Thus, track-side acceleration sensor systems that are specifically dedicated to condition monitoring of the crossing (cf. [29]) and that are mounted directly to or close to the crossing seem justified. Acceleration data measured at this point of the switch are naturally dominated by the impacts of wheels on the crossing. Given the experimental results so far, a combination of such a system with an acceleration sensor mounted to other positions on the switch, e.g., at the point machine, would be recommendable.

### 3.7. Comparison

**Figure 16:**
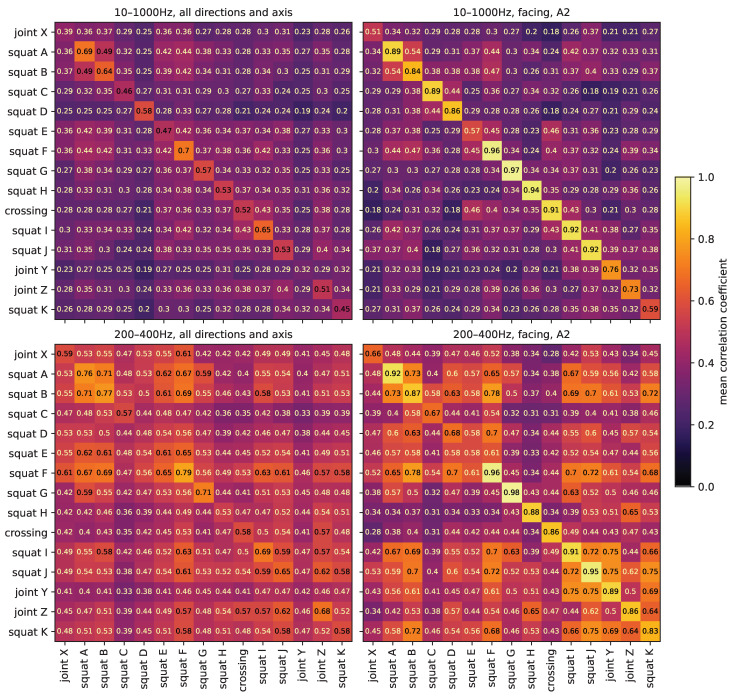
Mean correlation coefficient between impact event time series of the track discontinuities and defects in the experiment. For the top plots, the data are band-pass filtered at 10–1000 Hz after standard pre-processing, for the bottom plots at 200–400 Hz. The plots on the left contain all combinations of all driving directions and axle, the plots on the right only in the facing direction and axle A2. For all plots, the maximum allowed timelag is set to 0.01 s, to compensate for the manually labeled start times.

To compare the different track discontinuities and defects in the experiment, the waveform of their impact events are directly compared using the correlation coefficient. Figure 16 shows the mean correlation coefficient between impact event time series of the track discontinuities and defects in the experiment. For the top plots, the data are band-pass filtered to 10–1000 Hz, for the bottom plots to 200–400 Hz. The plots on the left contain all combinations of all driving directions and axle, the plots on the right only in the facing direction and axle A2. In each plot, the diagonal contains the average of correlation coefficients between different impact events caused by the same origin. The non-diagonal elements are the average of correlation coefficients between impact events caused by two different origins. For 10–1000 Hz, all driving directions and axles, the average correlation coefficient between different impact events caused by the same origin is 0.53 (min 0.12, max 0.98), while the average correlation coefficient between impact events caused by two different origins is 0.32 (min 0.08, max 0.75). The corresponding values for facing direction and axle A2 only are 0.81 (min 0.39, max 0.98) and 0.31 (min 0.10, max 0.63). Thus, again, the direction and axle have a strong impact on the waveforms, but if both are kept the same, impact events caused by the same origin are highly reproducible. This is in agreement with the previously presented results. For 200–400 Hz, all driving directions and axles, the average correlation coefficient for impact events of the same origin is 0.62 (min 0.13, max 0.99) and that of different origins is 0.50 (min 0.09, max 0.91). The corresponding values for facing direction and axle A2 only are 0.83 (min 0.51, max 0.99) and 0.51 (min 0.16, max 0.86). Filtering the data to a narrow frequency band around 300 Hz, all impact events have more similar waveforms, regardless of origin, direction or axle. Differentiation by individual origin becomes increasingly difficult, even for only one direction and axle.

A common detection method for squats with axle box acceleration data in the literature is to detect spikes in the power of predefined frequency bands, e.g., [7] with frequencies around 300 Hz and 1060–1160 Hz or [8] with frequencies around 300 Hz and 1000–2000 Hz. These frequencies bands are characteristic frequencies of the track and can depend on a number of factors, including the position of a squat relative to the closest sleepers [10]. This, to some extent, also works in the experiment setup [38,39] for frequencies around 300 Hz. These studies are solemnly aimed at squats, and differentiation toward other types of impact event origins or the overlap of various impact events at the same time is not discussed. However, in the experimental data, both in the case of broadband (10–1000 Hz) and narrowband (200–400 Hz) signals, waveform correlation cannot serve to group the data by track discontinuity/fault type (i.e., squat, joint or crossing). This is in agreement with the results from Section 3.5. Filtering the data to a narrowband around 300 Hz only serves to decrease the differences between events and could be beneficial for the detection of any type of impact, but not to differentiate between them. Here, for the facing direction and axle A2, some track discontinuity/faults that are located close to each other have a higher correlation with each other, regardless of their type, e.g., squat I, squat J and joint Y. This is not the case for the broadband signal. In a single sensor setup, impacts will naturally be at some distance from the sensor. At the same time, the transmission functions between impact event origins and sensor are frequency-dependent and hard to calculate, due to the complex layout of a switch, as discussed in Section 3.1. Essentially, the signal transmission function to the sensor is different for each impact event origin location. Methods that use broadband signals seem to be particularly susceptible to these influences, while filtering to a narrowband could negate this effect to some extent.

All in all, this suggests that, in practice, narrowband signals could potentially be used to detect impact events, but the discrimination between impact event origins requires broadband signals. However, depending on train speed and the number of track discontinuities and defects in a switch, a number of impact events occur at the same time, as seen in Section 3.2. The implications of this remain to be seen.

## 4. Conclusions and Future Research

### 4.1. Conclusions

There has been very little research into the monitoring of local track defects and discontinuities in railway switches using track-side acceleration sensors. This paper presents a novel study of transient impact events in an experimental setup of a switch with track-side acceleration sensors, using frequency and waveform analysis. All impact events in the experiment, i.e., the full impact event signature, could be labeled and showed repeatable waveforms and frequency content when measurements were taken in the same configuration. The waveform and frequency content of impact events are influenced by a number of factors. The main influence on the impact event signals is the signal transmission from the impact event source to the sensor, which is determined by their relative locations. Even if all other factors are held constant, this will dominate the waveform and frequency content of the recorded signal, overpowering the influence of the type of track discontinuity/fault. Due to the small variation in bogie speed and its correlation with other factors, the influence of the bogie speed could not be analyzed. No unique frequencies or waveform characteristics could be found for each type of track discontinuity or fault. This is a significant difference from the findings on axle box acceleration data in the literature, where the transmission between the track discontinuity/fault and the sensor is the same for all impact events and the frequency content can be used for diagnostic purposes. As previously suggested for axle box acceleration data and in the context of this experiment, narrowband signals around certain natural frequencies of the switch could potentially be used to detect impact events in general. However, signals with a wider frequency bandwidth are required to distinguish between individual impact events caused by different track discontinuities/defects and axles. This could help to later identify the type of track discontinuity/fault, i.e., to perform a diagnosis, as seen in the next subsection. The experimental setup provides a controlled environment, but it also has some general limitations, e.g., very low speed and bogie weight, which influence the results compared to field data. In practice, the impact event signature of a train passing a switch contains a number of overlapping events. It remains to be seen how this affects the detection and diagnosis of impact events.

### 4.2. Future Research

Based on the results presented, a single track-side acceleration sensor setup is not recommended to monitor local track discontinuities and defects distributed over an entire railway switch. Instead, the combination of several time-synchronized acceleration sensors along a switch into a multi-sensor system seems to be more promising. Of these, at least one acceleration sensor should be placed at the point machine (for dual monitoring purposes), and one at the crossing. Full sensor coverage would also make it possible to monitor the ballast [25]. Alternatively, track-side sensors that naturally span over long distances could be considered, e.g., fiber optic sensors. Aside from that, for train-side monitoring solutions, axle box acceleration sensors appear to be a promising approach. Existing diagnostic algorithms would need to be tailored to switches and tested against a labeled dataset of clusters of several local track defects and discontinuities in close proximity.

More labeled (field) data are needed to continue research on monitoring local track discontinuities and defects with track-side acceleration sensors in switches. A next step could be to record the track-side accelerations of trains passing over several switches (with local track defects) over an extended period of time. Additionally, train speeds, axle spacing, axle detection at a fixed point on the switch, and the exact layout of the switches with all their discontinuities and defects should be collected.

Such a dataset could be used to further develop both data-driven and physical approaches. For data-driven approaches, based on the literature and the results of this paper, the following steps would be recommended. Group train passages by train type, direction and speed and pre-calculate their impact event signatures. Then explore common methods for the detection and diagnosis of impact events and test them against the ground truth (i.e., pre-calculated impact event signature). For the detection of impact events, narrowband signals are preferable, while broadband signals are needed to match individual impact events from multiple train passages, differentiated by axle and impact event source. Monitor for changes in the observed impact events over time, e.g., in amplitudes, waveforms, frequency content or new additional impacts. One of the major challenges in real field data will be the overlap of impact events (see Figure 6) and the number of other simultaneous vibrations.

## Figures and Tables

**Table 1:** Sensor specifications and setup for the data considered in this study.

Sensor		Setup	
Model	608A11 industrial ICP^®^ accelerometer	Direction	vertical
Axis	single-axis	Sampling rate	51,200 Hz
Sensitivity	100 mV/g		
Measurement range	±50 g		
Frequency range (±3 dB)	0.5 to 10,000 Hz		

## Data Availability

The data are available at: Reetz S.; Najeh T.; Lundberg J. Track-side accelerations of a bogie passing over a railway switch in an experimental setup. Zenodo, https://doi.org/10.5281/zenodo.10209428, accessed on 8 January 2024.
